# Impact of Genetic Risk Score and Dietary Protein Intake on Vitamin D Status in Young Adults from Brazil

**DOI:** 10.3390/nu14051015

**Published:** 2022-02-28

**Authors:** Buthaina E. Alathari, Nathália Teixeira Cruvinel, Nara Rubia da Silva, Mathurra Chandrabose, Julie A. Lovegrove, Maria A. Horst, Karani S. Vimaleswaran

**Affiliations:** 1 Hugh Sinclair Unit of Human Nutrition, Department of Food and Nutritional Sciences, University of Reading, Harry Nursten Building, Pepper Lane, Reading RG6 6DZ, UK; b.e.a.a.alathari@pgr.reading.ac.uk (B.E.A.); j.a.lovegrove@reading.ac.uk (J.A.L.); 2 Department of Food Science and Nutrition, Faculty of Health Sciences, The Public Authority for Applied Education and Training, P.O. Box 14281, AlFaiha 72853, Kuwait; 3 Nutritional Genomics Research Group, Faculty of Nutrition, Federal University of Goiás (UFG), Goiânia 74690-900, Brazil; nathaliateixeira@discente.ufg.br (N.T.C.); nutrinara@discente.ufg.br (N.R.d.S.); 4 Department of Psychology and Clinical Language Sciences, University of Reading, Harry Pitt Building, Earley Gate, Reading RG6 6ES, UK; m.e.chandrabose@student.reading.ac.uk; 5 Institute for Food, Nutrition and Health, University of Reading, Reading RG6 6AH, UK; 6 Institute of Cardiovascular and Metabolic Research, University of Reading, Reading RG6 6AA, UK

**Keywords:** 25(OH)D, genetic risk score, gene–diet interaction, nutrigenetics, protein intake, Brazil

## Abstract

Given the relationship between vitamin D deficiency (VDD) and adverse outcomes of metabolic diseases, we investigated the interplay of dietary and genetic components on vitamin D levels and metabolic traits in young adults from Brazil. Genetic analysis, dietary intake, and anthropometric and biochemical measurements were performed in 187 healthy young adults (19–24 years). Genetic risk scores (GRS) from six genetic variants associated with vitamin D (vitamin D-GRS) and 10 genetic variants associated with metabolic disease (metabolic-GRS) were constructed. High vitamin D-GRS showed a significant association with low 25(OH)D concentrations (*p* = 0.001) and high metabolic-GRS showed a significant association with high fasting insulin concentrations (*p* = 0.045). A significant interaction was found between vitamin D-GRS and total protein intake (g/day) (adjusted for non-animal protein) on 25(OH)D (*p*_interaction_ = 0.006), where individuals consuming a high protein diet (≥73 g/d) and carrying >4 risk alleles for VDD had significantly lower 25(OH)D (*p* = 0.002) compared to individuals carrying ≤4 risk alleles. Even though our study did not support a link between metabolic-GRS and vitamin D status, our study has demonstrated a novel interaction, where participants with high vitamin D-GRS and consuming ≥73 g of protein/day had significantly lower 25(OH)D levels. Further research is necessary to evaluate the role of animal protein consumption on VDD in Brazilians.

## 1. Introduction

An increased prevalence of vitamin D deficiency (VDD) has been reported worldwide [1,2,3]; however, in South America, studies reporting the prevalence of vitamin D deficiency are scarce [4,5,6]. Brazil, the largest country in South America, has a low-latitude and elevated ultraviolet rays (UVB) [7] and mostly tropical weather conditions with high temperatures throughout the year [8]. Despite having abundant year-round sunshine, vitamin D deficiency and insufficiency have been reported by recent studies to be at 28.2% and 45.3%, respectively, regardless of age or gender [5]. Dietary consumption of vitamin D is generally low in the Brazilian populace and the amount of vitamin D in foods in Brazil is not accurately known. Moreover, many food products do not list information about vitamin D quantity in the food composition table and this hinders any studies trying to assess the influence of dietary intake of vitamin D on serum 25(OH)D levels. In general, dietary sources of vitamin D are limited and the small bioavailable amounts found in foods are inadequate for the phycological requirements of the human body [5,9].

In Brazil, overweight and obesity are important health concerns. Overweight was reported at 43% in 2006, 53.8% in 2016 [10] and has increased in prevalence to 57.5% in 2020 [11]. Obesity was reported at 11.8% in 2006, increased to 18.9% in 2016 [10] and increased in prevalence to 21.5% in 2020 in the Brazilian population [11]. The concern over the increased frequency of overweight and obesity is due to their poor health consequence and being a key risk factor to several chronic diseases such as diabetes, cardiovascular disorders and some types of cancer [12]. Type 2 diabetes (T2D) is another health issue of concern in the Brazilian population and the country is ranked 5th in the world for highest number of people with T2D [13]. Furthermore, a little over 75% of T2D patients are either overweight or obese [14].

Several epidemiological studies demonstrated a relationship between low vitamin D status and metabolic traits [15,16,17,18]. Using genetic polymorphisms to explore the relationship between VDD and metabolic diseases can help minimize the inherent limitations of nutritional epidemiological studies [19,20]. The use of genotypic information limits the effect of residual confounding from unconsidered factors or uncollected data [21]. Given that there are limited nutrigenetic studies in the Brazilian young adult population, this study will examine the association of the vitamin D-related genetic risk score (GRS) and metabolic disease-related GRS with clinical, biochemical and anthropometric parameters [22,23], and examine interactions between the GRSs and lifestyle factors on metabolic disease outcomes in young Brazilian adults using a nutrigenetic approach.

## 2. Methodology

### 2.1. Study Population

Obesity, Lifestyle and Diabetes in Brazil (BOLD) is a cross-sectional study in young healthy Brazilian adults aged 19–24 years enrolled at the Federal University of Goiás (UFG) between the months of March and June 2019. The study took place as part of Gene–Nutrient Interactions (“GeNuIne” Collaboration) which is an ongoing collaboration aiming to examine the effect of genes and lifestyle on chronic diseases in ethnically diverse populations [24,25,26,27]. A baseline questionnaire was completed by all participants regarding health status, socioeconomic, and demographic status. Exclusion criteria included use of hypoglycemic or lipid-lowering drugs; use of vitamin or mineral supplements; undergoing any dietary intervention in the past 6 months; engaging in vigorous physical activity; having acute medical conditions such as fever, inflammation, infection, diarrhea, or being diagnosed with chronic diseases such as hypertension, diabetes mellitus, rheumatoid arthritis, cancer, or cardiovascular disease. In total, 200 individuals completed the BOLD study; however, for the present analysis after excluding 13 participants with incomplete genetic data the total number of participants included was 187. Ethical approval was granted by the Ethics Committee of the Federal University of Goiás (protocol number 3.007.456, 8 November 2018), and performed according to the ethical principles in the Declaration of Helsinki. All study participants signed an informed consent form.

### 2.2. Anthropometric Measures

Anthropometric measurements were taken by trained investigators using validated methods. Measurements of body weight, height and waist circumference (WC) were taken using identical techniques [28,29]. A Tanita^®^ (Tanita Corporation, Itabashi, Tokyo, Japan) portable electronic scale was used to weigh participants, the maximum capacity was 150 kg. Height measurements were taken using a stadiometer with a mobile rod. Measurements of WC were obtained by using a non-stretchable measuring tape. Calculation of body mass index (BMI) was performed using the following formula: weight (kg)/height (m^2^). Dual Energy Radiological Absorptiometry scan (DXA) was used to measure body composition, using the Lunar DPX NT model (General Electric Medical Systems Lunar^®^; Madison, WI, USA). Body fat percentage (BFP) was considered elevated when it was 30.0% for women and 20.0% for men [30,31,32].

### 2.3. Biochemical Measures

Blood samples were collected by a qualified professional, through a peripheral venous puncture in the morning after a 12 h fast (not eating or drinking anything but water), with additional advice not to consume alcohol 72 h before blood collection. Ethylene-diamine-tetra acetic acid (EDTA) tubes using a BD Vacutainer^®^ were used to collect blood samples and the samples were immediately processed after pooling at Romulo Rocha Laboratory (Goiânia, Brazil) to obtain plasma and serum, respectively. Serum vitamin D concentration was measured by chemiluminescence, using the model Architect i1000, Abbott Diagnostics [33]. The fasting blood glucose and insulin were analyzed by the enzymatic colorimetric technique, with an automatic System Vitros Chemistry 950 XRL (Johnson & Johnson, New Brunswick, NJ, USA). Glycated hemoglobin (HbA1c) measurements were undertaken using high-pressure liquid chromatography (HPLC-Bio-Rad Laboratories, Hercules, CA, USA).

### 2.4. Assessment of Sun Exposure and Dietary Intake

Our study was conducted in the state of Goiás, which is in the central-west region of Brazil, during the fall season between the months of March and June 2019 where the average annual temperature is 24.6 °C [34] and the sunlight hours range between 11 and 12 h [35]. Sun exposure was assessed using a specific validated questionnaire adapted from the MIT-UV study protocol, which includes the usual time of daily exposure, commonly exposed body parts, use of sunscreen and factor (SPF) and skin type [36]. Daily sun exposure was recorded for 7 days and determined in 3 categories and assigned a numerical value: 1 = under 5 min of sun exposure; 2 = between 5 and 30 min of sun exposure; 3 = over 30 min of sun exposure. Total sun exposure for the week was determined by summing up the numerical values for each day which resulted in a range of 7–21 representing sun exposure for each participant. A median value of 16 was used to define participants, where individuals with value <16 and ≥16 were categorized as those with low and high sun exposures, respectively [36].

Intakes of food were assessed by experienced nutritionists using a three-day food diary (two weekdays and one weekend day) [37]. Food measuring equipment such as measuring spoons and measuring cups were provided to participants to aid them in approximating portion sizes of foods. To determine exact amounts of consumed foods the software Avanutri Online^®^ diet calculation (Avanutri Informática Ltd., Rio de Janeiro, Brazil) was used to convert food intakes into grams. The protein consumption was categorized as animal (including all kind of meat, fish, eggs, milk, and dairy products), and non-animal protein source in grams.

### 2.5. SNP Selection and Genotyping

We selected a total of 16 SNPs, of which 6 SNPs previously showed associations with vitamin D levels and 10 SNPs previously showed associations with metabolic traits in several ethnic groups. Six vitamin D-related SNPs were included in the analysis: vitamin D receptor (*VDR*) SNPs rs2228570 and rs7975232 [38,39]; 7-dehydrocholesterol reductase (*DHCR7*) SNP rs12785878 [40,41,42,43]; 25-hydroxylase (*CYP2R1*) SNP rs12794714 [43,44]; 24-hydroxylase (*CYP24A1*) SNP rs6013897 [41,45]; and vitamin D binding protein (*DBP*)/group-specific component (*GC*) SNP rs2282679 [40,44]. Ten metabolic disease-related SNPs were included in the analysis: Fat mass and obesity-associated (*FTO*) SNPs rs8050136 and rs9939609 [20,46,47,48,49,50,51]; transcription factor 7-like 2 (*TCF7L2*) SNPs rs12255372 and rs7903146 [20,52,53,54,55,56,57,58]; melanocortin 4 receptor (*MC4R*) SNP rs17782313 [20,59,60,61,62]; potassium voltage-gated channel subfamily Q member 1 (*KCNQ1*) SNPs rs2237895 and rs2237892 [63,64]; cyclin dependent kinase inhibitor 2A/B (*CDKN2A*/B) SNPs rs10811661 [53,65,66,67]; Peroxisome Proliferator Activated Receptor Gamma (*PPARG*) SNP rs1801282 [53,68,69,70]; calpain 10 (*CAPN10*) SNP rs5030952 [71,72].

DNA analysis was performed by collecting blood samples (3 mL each) in EDTA tubes BD Vacutainer^®^ tubes which were transferred in a (−80 °C) temperature-controlled environment by the World Courier Company to perform genotyping at the LGC Genomics, London, UK (http://www.lgcgroup.com/services/genotyping, accessed on 26 February 2022).

### 2.6. Statistical Analysis

Statistical software SPSS (v27; SPSS Inc., Chicago, IL, USA) was used to conduct statistical analyses. The selected 16 SNPs were in Hardy–Weinberg equilibrium (HWE) (*p* > 0.05), which was tested using a goodness-of-fit chi square test (Supplementary Table S1). Descriptive features of study population were given as means and standard deviations (SD) for continuous variables and comparisons between groups were tested using independent samples t-test. Shapiro–Wilk test of normality was conducted on all continuous variables to verify the normality of the data in the variables. Log-transformation was performed on all non-normally distributed variables, and these variables included age (years), BMI (kg/m^2^), waist circumference (WC) (cm), glucose (mg/dL), insulin (nmlU/L), HbA1c (ng/mL), total energy intake (Kcal), total carbohydrate (g), total protein, animal protein, non-animal protein (g), total fat (g), fiber (g), and 25(OH)D level (ng/mL).

Vitamin D cut-offs were decided based on the 2020 revised reference values of ‘The Brazilian Society of Endocrinology and Metabolism and The Brazilian Society of Clinical Pathology/Laboratory Medicine’. The vitamin D values for the general Brazilian population were defined as normal when 25(OH)D levels were between 20 and 60 ng/mL, and deficient when 25(OH)D levels were below 20 ng/mL [73].

**Figure 1:**
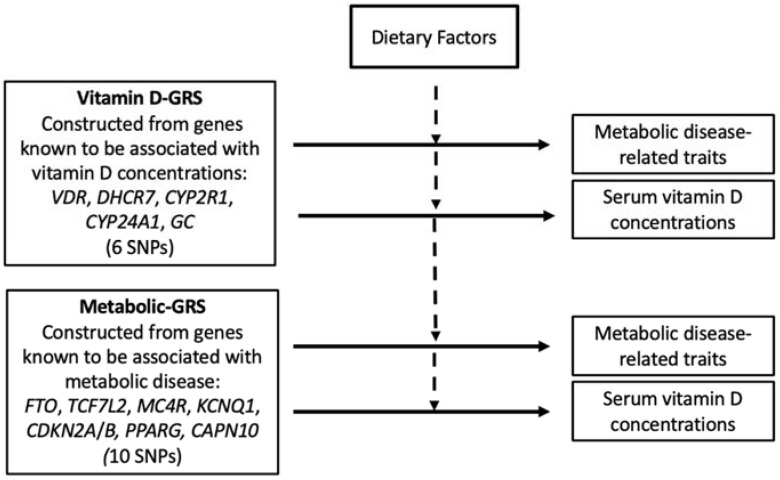
Study design. The one-sided horizontal arrows with solid lines represent the genetic associations and the one-sided vertical arrows with dotted lines represent the interactions between GRS and diet on clinical and biochemical measurements. The association of vitamin D-GRS with 25(OH)D levels and metabolic traits and the association of the metabolic-GRS with 25(OH)D levels and metabolic traits and were tested. Furthermore, analyses of the effect of dietary factors on these genetic associations were performed. Abbreviations: GRS: genetic risk score; SNP: single nucleotide polymorphism; *VDR*: Vitamin D Receptor; *DHCR7*: 7-dehydrocholesterol reductase; *CYP2R1*: 25-hydroxylase; *CYP24A1*: 24-hydroxylase; *GC*: group-specific component; *FTO*: fat mass and obesity-associated gene; *TCF7L2*: transcription factor 7-like 2 gene; *MC4R*: melanocortin 4 receptor gene; *KCNQ1*: potassium voltage-gated channel subfamily Q member 1; *CDKN2A/B*: cyclin dependent kinase inhibitor 2A/B; *PPARG*: Peroxisome Proliferator Activated Receptor Gamma; *CAPN10*: calpain 10.

Two independent genetic risk scores (GRSs) were created by the addition of the sums of the risk allele across each SNP. The vitamin D-GRS was computed from six SNPs and the metabolic disease GRS was computed from 10 SNPs. Each SNP had a value of 0, 1, or 2 and this value indicates the number of risk alleles for each GRS. Subsequently, these values were calculated by adding the number of risk alleles across each SNP. For each individual GRS, risk allele scores were then divided by the median and categorized into a “low genetic risk group” and a “high genetic risk group.” Using the median of vitamin D-related GRS, low risk and high risk were categorized as individuals carrying ≤ 4 (*n* = 112) and those carrying > 4 (*n* = 71) risk alleles, respectively. For the metabolic disease- GRS, low risk relates to individuals carrying ≤ 5 (*n* = 123) and high risk relates to those carrying > 5 (*n* = 60) risk alleles. Figure 1 denotes the study design of the performed analyses.

Linear regression models were used to analyze the association of vitamin D-related GRS and the metabolic disease-related GRS on anthropometric and biochemical outcomes (BMI, WC, 25(OH)D, glucose, HbA1c, fasting insulin), respectively. Additionally, the interaction between GRSs and dietary factors on clinical and biochemical variables were tested using linear regression models by including the interaction term (GRS*dietary factor). Regression models were adjusted for age, BMI, sun exposure and total energy intake, wherever appropriate. Dietary factors included total carbohydrate, total protein (animal protein, non-animal protein), total fat, and fiber intake in grams. Furthermore, in cases where interactions were statistically significant, study participants were split by the tertiles of dietary consumption and further analyzed.

## 3. Results

### 3.1. Characteristics of Participants

Anthropometric, biochemical, and dietary parameters of the BOLD study participants were compared based on 25(OH)D status and are summarized in Table 1. No significant differences were found between participants with normal 25(OH)D levels and participants with 25(OH)D deficiency except for total protein intake (g) (*p* = 0.008).

### 3.2. Association between Vitamin D-GRS and Anthropometric and Biochemical Measurements

**Figure 2:**
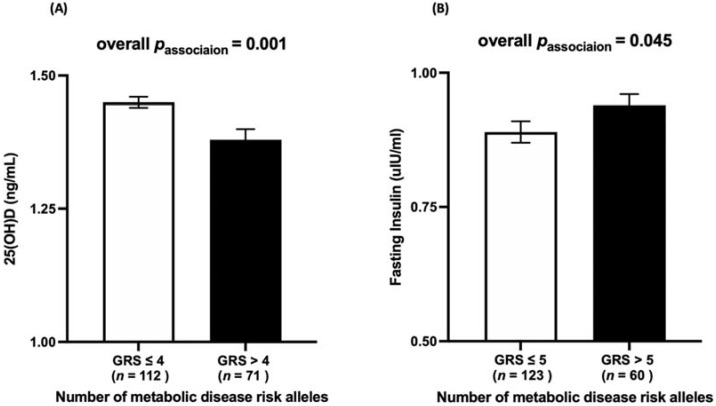
(**A**) The association between vitamin D-GRS and log 25(OH)D. Participants carrying >4 vitamin D risk alleles (mean ± SE: 1.38 ± 0.02) had lower 25(OH)D levels compared to participants with ≤4 risk alleles (mean ± SE: 1.45 ± 0.01). (**B**) The association between metabolic-GRS and log fasting insulin. Participants carrying >5 metabolic risk alleles (mean ± SE: 0.94 ± 0.02) had higher fasting insulin levels compared to participants with ≤5 risk alleles (mean ± SE: 0.89 ± 0.02).

There was a statistically significant association between vitamin D-GRS and serum 25(OH)D concentrations (*p* = 0.001). Individuals who carried >4 vitamin D risk alleles (mean ± SE: 1.38 ± 0.02) had significantly lower 25(OH)D levels compared to participants carrying ≤4 risk alleles (mean ± SE: 1.45 ± 0.01) (Figure 2A).

### 3.3. Association between Metabolic-GRS and Anthropometric and Biochemical Measurements

A statistically significant association was observed between metabolic-GRS and fasting insulin (*p* = 0.045), where individuals who carried >5 metabolic risk alleles (mean ± SE: 0.94 ± 0.02) had significantly higher fasting insulin levels compared to individuals with ≤5 risk alleles (mean ± SE: 0.89 ± 0.02) (Figure 2B).

### 3.4. Interaction between the Vitamin D-GRS and Dietary Factors on Biochemical and Anthropometric Measurements

**Figure 3:**
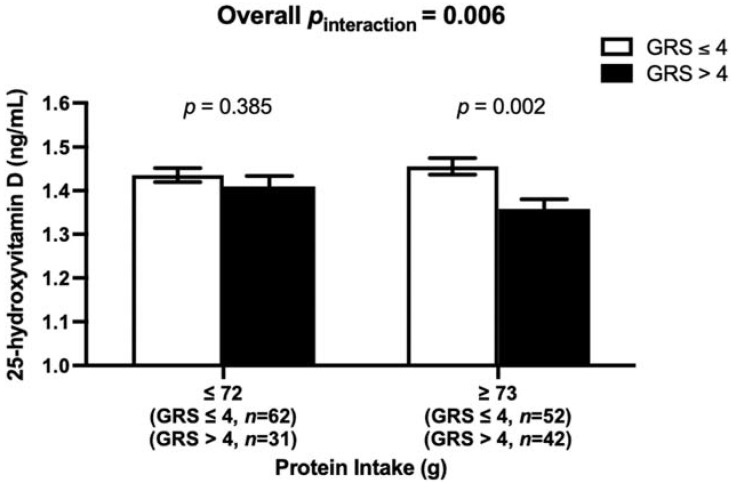
Interaction between vitamin D-GRS and total dietary protein intake (g) on log 25(OH)D (*p*_interaction_ = 0.006) adjusted for non-animal protein intake. Participants who had high protein intake (≥73 g/day) and GRS > 4 (mean ± SE 1.36 ± 0.021) had significantly lower 25(OH)D (*p* = 0.002) than participants with GRS ≤ 4 (mean ± SE: 1.46 ± 0.019).

A statistically significant interaction was found between the vitamin D-GRS and total protein intake (g) on log 25(OH)D concentrations (*p*_interaction_ = 0.006), where participants who had high protein intake (≥73 g/d) and >4 risk alleles, had significantly lower log 25(OH)D concentrations (mean ± SE 1.36 ± 0.021, *p* = 0.001) than participants with ≤4 risk alleles (mean ± SE: 1.46 ± 0.019, *p* = 0.001). Even after adjusting for non-animal protein sources, the interaction of vitamin D-GRS with protein intake (g) was statistically significant, where a participants with >4 risk alleles had significantly lower log 25(OH)D concentrations (mean ± SE 1.36 ± 0.021, *p* = 0.002) than participants with ≤4 risk alleles (mean ± SE: 1.46 ± 0.019, *p* = 0.002), (Figure 3). In addition, there was also a significant interaction of vitamin D-GRS with total protein intake (g) on BFP (*p*_interaction_ = 0.049), and with fat intake (g) on fasting glucose (*p*_interaction_ = 0.019), and with MUFA intake (g) on fasting glucose concentrations (*p*_interaction_ = 0.027).

### 3.5. Interaction between the Metabolic-GRS and Dietary Factors on Clinical and Biochemical Measurements

There was no significant interaction between metabolic-GRS and dietary intake on serum 25(OH)D concentrations and clinical and biochemical factors (*p* > 0.079 for all comparisons), respectively (Supplementary Table S2).

## 4. Discussion

As far as we know, this study is the first to examine the link between vitamin D status and metabolic disease-related traits using a nutrigenetic approach in young healthy Brazilian adults. Our study demonstrated the association of the high vitamin D-GRS with lower serum vitamin D concentrations and the association of metabolic-GRS with fasting insulin concentration. The main finding of this study is a significant interaction between the vitamin D-GRS and total protein intake on serum vitamin D levels after adjusting for non-animal protein intake. The association of total protein intake in participants with higher genetic risk of VDD on 25(OH)D levels is interesting; further investigation of the impact of higher protein intakes (from animal and non-animal sources) on vitamin D status using a randomized controlled trial would be helpful to illustrate whether this is a true finding.

In our study, we generated a GRS based on six vitamin D-related SNPs in genes involved in vitamin D metabolism. The vitamin D-GRS was associated with low vitamin D levels indicating that it is an ideal instrument for vitamin D deficiency. Even though we were unable to provide statistical evidence for the association between genetically instrumented vitamin D-GRS and metabolic disease traits in our study, we identified that individuals who had higher genetic risk alleles (GRS > 4) and consumed higher amounts of protein (≥73 g/day) had significantly lower 25(OH)D than participants with lesser risk alleles (GRS ≤ 4). Studies looking into the effect of dietary intake in people with high genetic risk for vitamin D deficiency are uncommon and studies reporting on the effect of dietary protein intake on vitamin D status for individuals with high genetic risk of vitamin D deficiency are non-existent. We previously investigated the effect of genetic factors and dietary intake in the Indonesian Minangkabau women and discovered a significant interaction between dietary carbohydrate intake and high vitamin D genetic risk on body fat composition (*p*-interaction = 0.049) [19]. This implicates the significance of genetic and dietary heterogeneity that exists across multiple ethnic groups. A randomized weight-loss intervention trial [74] in 118 overweight and obese participants in the United States, where the participates were randomly assigned to a weight-loss diet for two years with different percentages of caloric energy from macronutrients, showed a significant interaction between vitamin D GRS and dietary fat intake on two-year changes in whole-body bone mineral density (*p*-interaction = 0.02). Nevertheless, this study did not examine vitamin D status [75]. In comparison to the previous studies, our findings are novel and hence further studies relating to vitamin D GRS–diet interactions on vitamin D status are necessary to corroborate our findings.

The mechanism of the possible effect of high protein intake on vitamin D concentrations particularly in genetically VDD susceptible individuals is not clear, however, it could be specifically driven by animal protein sources as the findings in our study remained significant after adjustments for non-animal protein sources. Our finding is contrary to the results from a couple of studies on bone and skeletal health where it was reported that high protein intake positively interacts with vitamin D metabolism through the production of insulin-like growth factor-I (IGF-I) and enhances renal production of 1,25 dihydroxyvitamin D [76,77]. Nevertheless, these studies did not specify the sources of the dietary protein that enhanced vitamin D metabolism. In a cross-sectional study investigating vitamin D concentrations in 176 healthy vegetarian vs. non-vegetarian Pakistani individuals, a significantly lower serum 25(OH)D (*p* = 0.001) was found in individuals who were non-vegetarians (*n* = 9; mean ± SD: 9.39 ± 2.45) compared to vegetarians (*n* = 83; mean ± SD: 13.78 ± 3.48) [78], but whether the reduced 25(OH)D levels were influenced by increased animal protein intake was not investigated. The effect of dietary animal protein intake on vitamin D concentrations requires future investigations to confirm or refute our findings and to determine the molecular mechanisms of action.

According to the 2008–2009 data from the Brazilian Household Expenditure Surveys (HES), dietary protein and fat intakes have increased in Brazil while carbohydrates content has decreased [79]. Increase in protein intake was due to increased consumption of animal flesh and animal products. The mean daily caloric intake from a nationwide cross-sectional survey using the HES data of 2008–2009 was estimated to be 1902 kcal. The total carbohydrate intake contributed to 56% of the total energy, total fat intake contributed to 27% of total energy and total protein intake contributed to 17% of total energy with animal protein providing 10% of the total energy intake [79]. According to internationally accepted dietary guidelines [80,81,82] the total daily recommended protein intake is between 10 and 15% of total daily energy intake; this translates into 48–71 g of daily protein intake from the estimated caloric intake of the Brazilian population (1902 kcal). In our study, the protein intake of our participants ranged between 73 and 217 g/day which is higher than the daily protein intake recommendations. Hence, adherence to the dietary protein intake recommendations may be an effective strategy to overcome the genetic risk of vitamin D deficiency in Brazilians.

The main strengths of the study are that it is the first nutrigenetics analysis to investigate interactions between vitamin D-GRS and metabolic related traits in a healthy young Brazilian population. A genetic approach is favorable to observational studies as the genotype is not modified by the disease and is free from confounding [21,83]. Additionally, the use of a GRS analysis instead of a single SNP analysis is a favorable approach as the GRS method has a greater statistical power in predicting genetic predisposition over the single-locus approach [22,84]. Furthermore, biochemical and anthropometric measures were determined using validated techniques by skilled staff which improved the precision of these estimates. However, some limitations need to be acknowledged. One of the main limitations of the study is the small sample size, nevertheless, significant gene–diet interactions were identified suggesting that the study is well powered. Another limitation is that dietary intake was assessed using three-day self-reported food records; despite being a validated and widely used method, we cannot discount the effect of reporting and recall bias. Furthermore, the Brazilian population is an admixture of many genetic ancestries from all over the world which makes it genetically heterogenous and could cause biased estimates of disease risk because of population stratification [85,86,87,88]. Finally, the study population included healthy young adults which may not be demonstrative of the total Brazilian population.

## 5. Conclusions

In conclusion, this study discovered a novel interaction between vitamin D-GRS and total protein intake on serum 25(OH)D levels after adjusting for non-animal protein intake in a young Brazilian adult population, where individuals with high GRS consuming more than 73 g of protein/day had significantly lower 25(OH)D levels. The finding is of particular interest for setting public health recommendations for preventing 25(OH)D deficiency in genetically susceptible young healthy Brazilian individuals given the increase in the dietary intake of animal protein in recent years [79]. Further investigations and randomized controlled trials are required to shed more light on the effect of increased animal protein intake on vitamin D levels, especially in individuals genetically susceptible to VDD to enable effective public health interventions to prevent VDD.

## Figures and Tables

**Table 1:** Baseline features of study partakers stratified by 25(OH)D status.

Characteristics of Study Participants	*n*	Normal Vitamin D Status 25(OH)D ≥ 20 ng/mL	*n*	Deficient Vitamin D Status 25(OH)D < 20 ng/mL	*p* Value
Age (years)	154	21.32 ± 1.71	31	21.35 ± 1.56	0.928
BMI (kg/m^2^)	154	23.01 ± 3.87	31	23.76 ± 5.66	0.370
WC (cm)	154	74.05 ± 11.89	31	76.60 ± 14.04	0.291
BFP (%)	154	33.76 ± 10.65	31	34.57 ± 11.05	0.702
Glucose (mg/dl)	156	86.74 ± 6.79	31	88.35 ± 7.29	0.235
HbA1c (%)	156	4.73 ± 0.26	31	4.72 ± 0.22	0.911
Fasting Insulin (uIU/mL)	156	8.72 ± 3.69	31	8.80 ± 4.09	0.911
Total Energy Intake (kcal)	156	1793 ± 591	31	2024.12 ± 676.96	0.054
Total Protein (g)	156	75.20 ± 28.17	31	90.43 ± 33.48	0.008
Total Carbohydrate (g)	156	230.67 ± 84.32	31	258.08 ± 99.59	0.111
Total Fat (g)	156	63.34 ± 23.43	31	70.017 ± 24.88	0.153
Dietary Fiber (g)	156	14.45 ± 8.48	31	16.39 ± 9.68	0.258

Data is presented as means ± SD, *p* values were calculated by using the independent t test. Vitamin D cut-off points were created on the recommendations of the Brazilian Society of Endocrinology and Metabolism and the Brazilian Society of Clinical Pathology/Laboratory Medicine vitamin D levels [73]. Abbreviations: BMI: body mass index; WC: waist circumference; BFP: body fat percentage; HbA1c: glycated hemoglobin.

## Data Availability

The datasets used and/or analyzed during the current study are available from the corresponding author on reasonable request.

## Supplementary Materials

The following supporting information can be downloaded at: https://www.mdpi.com/article/10.3390/nu14051015/s1, Table S1: Genotypic distribution and allelic frequencies of the single nucleotide polymorphisms that were used to create the genetic risk score [89]. Table S2: Interactions between vitamin D-GRS and dietary factors on clinical and biochemical measurements.

## Abbreviations

VDD: vitamin D deficiency; T2D: type 2 diabetes; WC: waist circumference; BMI: body mass index; HbA1c: glycated hemoglobin; *VDR*: vitamin D receptor; *DHCR7*: 7-dehydrocholesterol reductase; *CYP2R1*: 25-hydroxylase; *CYP24A1*: 24-hydroxylase; *DBP*: vitamin D binding protein; *GC*: group-specific component; *CASR*: calcium sensing receptor; *FTO*: fat mass and obesity-associated gene; *TCF7L2*: transcription factor 7-like 2 gene; *MC4R*: melanocortin 4 receptor gene; *KCNQ1*: potassium voltage-gated channel subfamily Q member 1; *CDKN2A/B*: cyclin dependent kinase inhibitor 2A/B; *PPARG*: peroxisome proliferator activated receptor gamma; *CAPN10*: calpain 10; GRS: genetic risk score; SNP: single nucleotide polymorphism; HWE: Hardy–Weinberg equilibrium; SD: standard deviation; BOLD: Obesity, Lifestyle and Diabetes; HES: Household Expenditure Surveys.
