# Supplementary material for: Impact of Genetic Risk Score and Dietary Protein Intake on Vitamin D Status in Young Adults from Brazil
Source: Nutrients. 2022 Feb 28;14(5):1015. doi: 10.3390/nu14051015 (PMC8912678; doi:10.3390/nu14051015)
Supplement: Supplementary file 1 [file nutrients-14-01015-s001.zip › nutrients-1594176-supplementary.pdf]

Supplementary Table S1. Genotypic and allelic frequencies of the single nucleotide polymorphisms that were used to create the genetic risk score.

	Single Nucleotide Polymorphisms	Nucleotide Change	Total	Common Homozygous	Heterozygote	Rare Homozygous	Hardy Weinberg Equilibrium *p* Value	Minor Allele Frequency
VDR	rs2228570	T/C	195	CC: 83	TC: 89	TT: 23	0.91	T= 0.35
	rs7975232	C/A	200	AA: 72	CA: 92	CC: 36	0.49	C= 0.41
DHCR7	rs12785878	G/T	200	TT: 48	GT: 106	GG: 46	0.40	G= 0.50
CYP2R1	rs12794714	A/G	199	GG: 72	GA: 95	AA: 32	0.94	A= 0.40
CYP24A1	rs6013897	A/T	200	TT: 115	TA: 73	AA: 12	0.93	A= 0.24
GC	rs2282679	C/A	199	AA: 134	CA: 59	CC: 6	0.87	C= 0.18
FTO	rs8050136	A/C	197	CC: 71	AC: 96	AA: 30	0.79	A= 0.40
	rs10163409	T/A	198	AA: 117	TA: 64	TT: 17	0.06	T= 0.25
TCF7L2	rs12255372	T/G	200	GG: 85	TG: 92	TT: 23	0.80	T= 0.35
	rs7903146	T/C	200	CC: 90	TC: 87	TT: 23	0.78	T= 0.33
MC4R	rs17782313	C/T	200	TT: 139	TC: 55	CC: 6	0.84	C= 0.17
KCNQ1	rs2237895	C/A	199	AA: 85	CA: 91	CC: 23	0.86	C= 0.34
	rs2237892	T/C	200	CC: 160	TC: 35	TT: 5	0.08	T= 0.11
CDKN2A/B	rs10811661	C/T	200	TT: 151	CT: 45	CC: 4	0.76	C= 0.13

PPARG	rs1801282	G/C	200	CC: 170	GC: 30	GG: 0	0.25	G= 0.08
CAPN10	rs5030952	T/C	200	CC: 128	TC: 66	TT: 6	0.47	T= 0.20

*dbSNP database: https://www.ncbi.nlm.nih.gov/snp/ [1]

Reference

1. National Center for Biotechnology Information. https://www.ncbi.nlm.nih.gov/snp/.

Supplementary Table S2. Interaction between metabolic-GRS and dietary factors on clinical and biochemical measurements.

Clinical and Biochemical Parameters	Carbohydrates (g) $p_{interaction}$	Protein (g) $p_{interaction}$	Fat (g) $p_{interaction}$	Fibre (g) $p_{interaction}$
25-hydroxyvitamin D (ng/ml)	0.999	0.525	0.602	0.208
BMI (kg/m2)	0.355	0.913	0.403	0.989
WC (cm)	0.997	0.349	0.839	0.734
BFP (%)	0.367	0.756	0.231	0.807
Glucose (mg/dl)	0.930	0.827	0.131	0.851
HbA1c (ng/ml)	0.694	0.392	0.438	0.792
Fasting Insulin (uIU/ml)	0.079	0.537	0.641	0.750

All variables were log transformed except for body fat percentage values which were in normal distribution. All associations were adjusted for age, gender, sun exposure, total energy intake and BMI (except BMI which was not adjusted for when the outcome was BMI). Abbreviations: GRS: genetic risk score, BMI: body mass index, WC: waist circumference, BFP: body fat percentage, HbA1c: glycated haemoglobin.
